# Evaluating the psychological mechanisms underlying new Olympic sports consumption: the expectation disconfirmation theory framework applied to Millennials’ and Generation Z’s experiences

**DOI:** 10.3389/fpsyg.2026.1766218

**Published:** 2026-02-11

**Authors:** Yong-Seok Jang, Ji-Young Choi, Sun-Young Lim

**Affiliations:** 1 Department of Physical Education, Kyung Hee University, Yongin-si, Gyeonggi-do, Republic of Korea; 2 Department of Physical Education, Seoul National University, Seoul, Republic of Korea

**Keywords:** 2024 Paris Olympic, consumer behavior, expectation disconfirmation theory, generation Z, mega sporting event, Millennials, new Olympic sports

## Abstract

**Introduction:**

This study investigated the psychological mechanisms underlying Millennials’ and Generation Z’s consumption of the newly adopted Olympic sports, breaking, skateboarding, surfing, and sport climbing, featured for the first time in the 2024 Paris Olympic Games. Drawing on the expectation disconfirmation theory (EDT), we examined how gaps between prior expectations and the actual viewing experience shape satisfaction and continuous viewing intentions.

**Methods:**

A survey was conducted from August to December 2024 involving Millennial and Generation Z respondents residing across 17 regions in Korea using purposive sampling to recruit individuals with prior viewing experience of the target sports. A total of 430 valid responses were analyzed in this study. The study was approved by the Institutional Review Board of Seoul National University (Approval Number: SNU 24-10-055) and complied with the established ethical research guidelines.

**Results:**

Findings indicated that prior expectations did not significantly influence disconfirmation or satisfaction, whereas perceived performance emerged as a central determinant of both constructs. Disconfirmation exerted a positive effect on satisfaction, suggesting that in contexts where prior information is limited, emotional and cognitive stimulation arising from performances exceeding expectations can enhance viewer satisfaction. Satisfaction significantly predicted continuous viewing intention.

**Conclusion:**

These results support the applicability of the EDT to Olympic sports content consumption while offering strategic implications for promoting sustained engagement among younger generations in newly introduced Olympic sports.

## 1 Introduction

The Olympic Games have evolved from traditional sporting competitions to a global cultural festival that reflects broader social changes. This transformation is largely due to the International Olympic Committee’s (IOC) ongoing efforts to adapt the Olympic sports program in line with shifting generational values, cultural dynamics, and societal expectations (Choi et al., 2022; International Olympic Committee, 2020b; International Olympic Committee, 2019; International Olympic Committee, 2023a; International Olympic Committee, 2023b; Tokyo Organising Committee of the Olympic and Paralympic Games, 2021). Younger cohorts, commonly referred to as Millennials and Generation Z, have begun to engage in sports not merely as a form of entertainment but as a means of achieving extreme accomplishment, self-expression, and identity formation through creative and challenging activities (Marques et al., 2025). Millennials include those born between the early 1980s and mid-1990s, whereas Generation Z includes those born between the late 1990s and early 2000s (Iorgulescu, 2016). Not all individuals within this cohort share the same characteristics; however, previous research has described Millennials and Generation Z as being highly familiar with digital environments and, compared with previous generations, more likely to value emerging trends, self-expressive activities, and personalized experiences (Marques et al., 2025; Iorgulescu, 2016; Lee and Jang, 2023).

This shift in consumption patterns highlights the growing disconnect between traditional Olympic sports and younger audiences’ experiential preferences. This misalignment exposes the limitations of long-established Olympic sports programs in appealing to emerging generations whose cultural orientation prioritizes individuality, innovation, and emotional resonance (Lim et al., 2025). In response to these evolving trends, the IOC implemented a strategic initiative to incorporate new sports, such as breaking, skateboarding, surfing, and sport climbing, into its official Olympic program, beginning with the Paris 2024 Games (International Olympic Committee, 2020a). These sports align closely with youth subcultures, action-based creativity, and lifestyle-oriented participation, thereby offering the potential to bridge the generational gap in Olympic engagement (Conti, 2023). In fact, these sports garnered significant engagement and positive responses from younger audiences when they were introduced as demonstration events at the Youth Olympic Games and the Tokyo Olympics (International Olympic Committee, 2018; Nam and Lim, 2024).

In this context, it is crucial to systematically analyze the expectations of younger generations regarding these sports and to assess whether their actual experiences meet, exceed, or fall short of these expectations. The long-term sustainability of the Olympics depends not on the institutional introduction of new sports but on the significance that these sports hold within the cognitive structures and experiential evaluations of the younger generation. Based on this recognition of the problem, this study adopted the expectation disconfirmation theory (EDT) as its analytical framework.

The theoretical framework in this study offers a novel and meaningful perspective for interpreting the turning points in the evolution of Olympic sports. Although this approach may be somewhat unconventional in Olympic studies, it offers crucial insights into how shifting generational sports consumption patterns reshape global sports governance and institutional strategies. Specifically, this study integrated the EDT to systematically analyze how Millennials and Generation Z form expectations about new Olympic sports, how their actual experiences align with or deviate from these expectations, and how this disconfirmation impacts their satisfaction, participation intentions, and sustained interest in the Olympic Movement. By doing so, we aimed to demonstrate the theoretical relevance and explanatory power of the EDT by connecting contemporary youth sports culture, characterized by creativity, self-expression, and challenge-oriented participation, with the IOC’s strategic inclusion of new sports, such as breaking, skateboarding, surfing, and sport climbing.

The EDT is a theoretical framework that explains the process by which satisfaction or dissatisfaction is formed through a comparison between a consumer’s prior expectations and actual experience (Shin, 2019). Tolman’s expectancy theory (Tolman, 1945), systematized by Oliver (1980), states that satisfaction is determined not by objective performance but by the degree to which perceived performance matches prior expectations (Oliver, 1997). In this study, expectations refer to the beliefs or predictions that consumers form about future outcomes or performance (Oliver, 2010), which are based on various sources of information such as an individual’s past experiences, the experiences of those around them, advertisements, and word of mouth (Kim and Chung, 2025).

Prior expectations—a core concept in consumer behavior theory—refer to the cognitive standards consumers use to predict the performance of a product or service (Kim and Hwang, 2019). After using or engaging with a product or service, consumers compare its actual performance with their prior expectations. During this process, consumers experience negative disconfirmation if performance falls short of expectations, positive disconfirmation if performance exceeds expectations, and simple congruence if performance meets expectations. These types of disconfirmation have a critical impact on the formation of satisfaction (Hien et al., 2024). Positive disconfirmation enhances satisfaction, attitude, repurchase intention, and loyalty, whereas negative disconfirmation decreases future engagement intentions and increases the likelihood of choosing a different option (Park et al., 2025). Therefore, the EDT can be utilized as a post-event evaluation model and an analytical tool to support practical decision-making in areas such as strategy development, customer retention, experience design, and brand management.

In this context, the EDT can be considered a representative consumer behavior theory that goes beyond simply explaining consumer satisfaction by clarifying the process by which satisfaction leads to attitudes, behaviors, loyalty, and sustainable relationships. Due to to its academic utility, the EDT is widely applied in various fields, including product and service consumption, tourism, sports, and cultural consumption (Kim and Chung, 2025; Hien et al., 2024; Park et al., 2025; Ahn et al., 2021; Li et al., 2020; Pitt et al., 1997). Nevertheless, prior research applying the EDT in the sports domain has primarily focused on the direct effects of expectation-performance disconfirmation on attitudes and behavioral outcomes (Park et al., 2025; Jeon and Lee, 2019; Oh et al., 2015; Kim and Lee, 2016). Consequently, relatively limited attention has been paid to structurally elucidating the cognitive and affective mechanisms through which the gap between expectations and actual experiences translates into satisfaction and subsequent continuance intentions.

Accordingly, this study aimed to empirically analyze how Millennials and Generation Z perceive and experience the new sports introduced at the 2024 Paris Olympics by applying the EDT. This study systematically measured, compared, and analyzed prior expectations, perceived performance based on actual viewing experience, and the discrepancy between them for new official sports such as breaking, skateboarding, surfing, and sport climbing. This study empirically investigated the impact of the gap between expectations and experiences on the overall satisfaction of the younger generation and their intention to continue participating in future Olympic Games. This study theoretically elucidates the mechanisms by which new sports enhance the long-term appeal and sustainability of Olympic sports in a changing sports consumption environment. The findings offer empirical and theoretical implications for content planning and operations that can strategically adapt the Olympics—as a global sports brand—to the changing preferences of different generations.

## 2 Theoretical background and hypotheses

### 2.1 Prior expectation, expectation disconfirmation, and satisfaction

Oliver (1980) defined prior expectations as consumers’ subjective evaluations of the likelihood of experiencing positive or negative outcomes when engaging in a particular behavior. Previous studies applying the EDT have primarily conceptualized prior expectations as anticipation of the performance of a product or service, and the present study adopted this conceptualization (Oliver, 1980; Zeithaml et al., 1993). Oliver and Burke (1999) suggested that prior expectations function as reference points within the EDT framework. The central assumption of the EDT is that consumer satisfaction increases when expectations are met or exceeded, whereas dissatisfaction emerges when perceived performance fails to meet prior expectations (Oliver, 1997; Oh et al., 2015; Oliver and DeSarbo, 1988). Therefore, within the EDT framework, the difference between a consumer’s prior expectations and actual experience based on multidimensional experience factors acts as an important determining factor in influencing satisfaction (Pitt et al., 1997; Jeon and Lee, 2019; Oh et al., 2015; Kim and Lee, 2016; Kim et al., 2010). In this context, Kim et al. (2010) argued that the gap between expectations and actual experiences can be more pronounced in unique and novel experiential environments, such as the introduction of a new Olympic sport. Furthermore, Parasuraman et al. (1988) reported that the gap between expectations and perceptions of service quality directly impacts customer satisfaction. We applied the results of these studies to the context of this study. This suggests that if prior expectations formed by Millennials and Generation Z regarding the new sports introduced at the 2024 Paris Olympics exceed their actual viewing experience, a positive discrepancy will occur, which is likely to lead to higher satisfaction. Accordingly, the following hypotheses are proposed:

*H1*: Prior expectations significantly affect expectation disconfirmation.

*H2*: Prior expectations significantly affect satisfaction levels.

### 2.2 Perceived performance, expectation disconfirmation, and satisfaction

Perceived performance refers to the evaluation that consumers form based on their actual experience after interacting with a product or service and serves as a key reference point for comparing prior expectations in judging satisfaction or dissatisfaction (Jeon and Lee, 2019). From an EDT perspective, disconfirmation and satisfaction are conceptualized as emotional responses derived from comparing perceived performance with prior expectations (Kim et al., 2004; Tse and Wilton, 1988; Fornell et al., 1996). In this context, the discrepancy resulting from a comparison between expectations and actual performance is categorized into three types: positive discrepancy (when actual performance exceeds expectations), negative discrepancy (when it falls short of expectations), and neutral discrepancy (when expectations and performance are aligned) (Oliver, 1981). The direction of this disconfirmation has been identified as a key determinant of satisfaction and has been shown to influence behavioral intentions such as revisits and recommendations (Hien et al., 2024; Ahn et al., 2021; Kim et al., 2010; Parasuraman et al., 1988; Kim et al., 2004; Tse and Wilton, 1988). This indicates that more favorable evaluations of actual experiences enhance consumers’ recognition of the gap between expectations and performance, leading to higher overall satisfaction. This relationship is expected to be salient in contexts with limited prior information and high experiential uncertainty, such as newly introduced sports events. Accordingly, we propose the following hypotheses:

*H3*: Perceived performance significantly affects expectation disconfirmation.

*H4*: Perceived performance significantly affects satisfaction.

### 2.3 Expectation disconfirmation and satisfaction

Expectation disconfirmation refers to the gap between consumers’ expectations of a product or service and their actual performance following their experience with it (Shin, 2019; Oliver, 1997; Park et al., 2025). When the perceived performance exceeds expectations, positive disconfirmation occurs, leading to increased consumer satisfaction (Oh et al., 2015; Kim and Lee, 2016; Kim et al., 2004). Conversely, when performance falls short of expectations, negative disconfirmation occurs, resulting in consumer dissatisfaction (Tolman, 1945; Oliver, 1980). In this study, satisfaction refers to consumers’ overall evaluation of their perceived experiences with a particular product or service (Fornell et al., 1996; Oliver, 1981). It is conceptualized as a psychological state arising from the discrepancy between prior expectations and actual experiences, encompassing both cognitive and emotional responses (Kim et al., 2004). When examined in the context of the EDT, consumer satisfaction increased further when positive disconfirmation occurred (Li et al., 2020; Pitt et al., 1997). Thus, Millennials’ and Generation Z’s expectation disconfirmation regarding the new Olympic events was expected to have a direct impact on satisfaction. Accordingly, this study proposes the following hypothesis:

*H5*: Expectation disconfirmation significantly affects satisfaction.

### 2.4 Satisfaction and intention to continue viewing

The intention to continue viewing is a psychological state in which consumers, after experiencing specific media or video content, engage in a holistic evaluation influenced by various factors (e.g., viewing satisfaction) and form an intention to continue consuming similar content (Jeon and Lee, 2019). Accordingly, this study conceptualized the behavioral intention of viewers who have watched a sporting event introduced as a new event at the 2024 Paris Olympics as wanting to continue watching it in the future (Oh et al., 2015; Nam and Lim, 2024). Oliver (1980) explained the relationship between customer satisfaction and continuance intention, arguing that satisfaction has a positive effect on consumers’ attitudes toward consumption, which has a significant effect on the intention to continue the relevant behavior. In addition, many studies have confirmed that consumer satisfaction positively affects both continuous consumption intentions and actual purchase behavior (Kim and Lee, 2016; Zeithaml et al., 1993; Oliver and Burke, 1999). This suggests that the satisfaction of Millennials and Generation Z viewers who watched the newly introduced sports games at the 2024 Paris Olympics could be an important factor in attracting potential Olympic viewers and increasing future viewership. Accordingly, we propose the following hypothesis:

*H6*: Satisfaction significantly affects the intention to continue viewing.

### 2.5 Hypothesized research model

**Figure 1:**
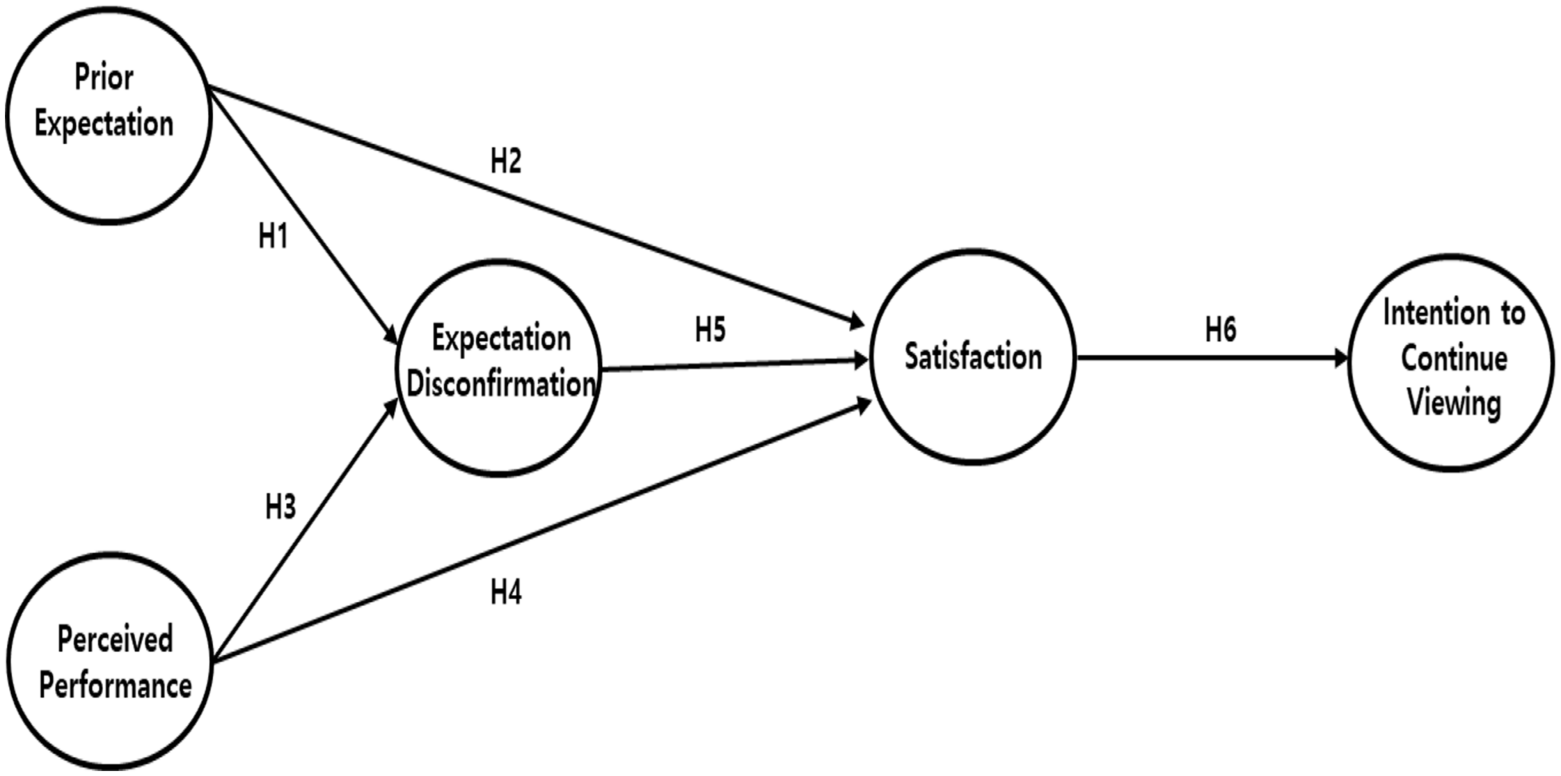
Research model.

The purpose of this study is to investigate the impact of discrepancies between Millennials’ and Generation Z’s preexisting expectations and actual viewing experiences regarding newly introduced Olympic sports on their satisfaction and continued viewing intentions. Based on the EDT proposed by Oliver and Burke (Oliver and Burke, 1999), we developed the research model as shown in Figure 1.

## 3 Methods

### 3.1 Participants

This survey was conducted from August 11, the day the 2024 Paris Olympics closed, to December 31. The participants were limited to Millennials and Generation Z, aged 19–30 years, living in 17 cities and provinces in the Republic of South Korea. This age range aligns with the strategic direction of the IOC, which aims to target the younger generation as its main target group (International Olympic Committee, 2023b; Oh et al., 2015). In fact, the IOC has adopted sports that match the interests and cultural sensibilities of the younger generation, such as breaking, skateboarding, surfing, and sport climbing, as official sports at the 2024 Paris Olympics to increase the younger generation’s interest in the Olympics and secure a long-term audience.

We employed purposive sampling, a non-probability sampling method, to identify participants who had experienced watching competitions in breaking, skateboarding, surfing, and sport climbing, which were introduced as new sports in the 2024 Paris Olympic Games. This aimed to increase the validity of the study and ensure the consistency of the analysis by selectively recruiting respondents with specific characteristics that fit the research purpose (Oliver and DeSarbo, 1988). The survey was conducted face-to-face, with the researcher visiting the survey site in person. Questionnaires were self-administered, and the respondents were asked to complete the questionnaire. Prior to the survey, all participants were fully informed of the purpose of the study and the processing of their personal information, and they provided informed consent. This study was approved by the Institutional Review Board (approval number: SNU 24-10-055) of Seoul National University, Republic of Korea, and was conducted in compliance with ethical research standards. A total of 445 questionnaires were distributed, of which 430 were selected and analyzed as the final valid sample after excluding 15 incomplete or insincere questionnaires (Table 1).

**Table 1:** Demographic characteristics of the participants.

Variables	Classification	Frequency (*n*)	Percentage (%)
Gender	Male	224	52.1
	Female	206	47.9
Age	10 s	122	28.4
	20 s	175	40.7
	30 s	133	30.9
Most favored new Olympic sports	Breaking	194	45.1
	Sport climbing	67	15.6
	Skateboarding	91	21.2
	Surfing	78	18.1
Frequency of viewing newly introduced Olympic sports during the Paris 2024 Olympic Games	Once	129	30.0
	2 times	248	57.7
	More than 3 times	53	12.3
Total		430	100

### 3.2 Measurement tool

The measurement tool used in this study was a structured questionnaire, and the measurement items used in previous studies related to the EDT were modified and supplemented to fit the purpose of this study. First, prior expectations and perceived performance comprised three items based on the scale developed by Oliver and Burke (Oliver and Burke, 1999) and referenced from the items used by Churchill and Surprenant (1982) and Yoon (2015). Second, satisfaction was measured using items extracted from Taylor et al. (2003) and Van Dolen et al. (2004), which were refined into three items. Third, the EDT scale was composed of three items based on the scale by Oliver (1997) and modified to fit this study based on the items used by Swan and Trawick (1981). Fourth, the intention to continue viewing included three items modified from those used by Bhattacherjee (2001) and Choi et al. (2009) to fit the purpose of this study. Finally, all questions, except for those on participants’ general characteristics (sex, age, most favored new Olympic sports, and frequency of viewing new Olympic sports), were measured using a 7-point Likert scale (1 = not at all, 7 = strongly agree). The 7-point Likert scale is considered one of the most optimal response formats in social science research, as it effectively captures neutral response options and subtle variations in attitudes without imposing an excessive cognitive load on the respondents (Preston and Colman, 2000). Accordingly, this study adopted the 7-point scale to enhance the reliability and sensitivity of the responses.

### 3.3 Validity and reliability of measurement

To ensure the intensive validity of the measurement tools (questionnaires) used in this study, content validity was verified by five expert groups consisting of two professors specializing in sports marketing and three PhD holders with expertise in sports marketing. Additionally, a confirmatory factor analysis (CFA) was performed to assess discriminant validity (Table 2).

**Table 2:** Confirmatory factor analysis.

Variables	Factors	*SC*	*SE*	*t*	C.R.	AVE	Cronbach’s *α*
Prior expectation	The newly introduced sports at the 2024 Paris Olympics were expected to contribute to enhancing the overall quality of the games.	0.896	—	—	0.885	0.842	0.877
	The new sports introduced at the 2024 Paris Olympics were expected to provide a positive experience of the games.	0.912	0.049	23.365			
	The new sports introduced at the 2024 Paris Olympics are expected to expand the diversity of sports and create a more interesting experience.	0.955	0.067	21.067			
Perceived performance	The new sports introduced at the 2024 Paris Olympics have increased the diversity of Olympic sports and brought about exciting and dynamic competitions.	0.812	—	—	0.799	0.642	0.847
	The new sports introduced at the 2024 Paris Olympics provided a positive Olympic experience.	0.891	0.052	21.899			
	The new sports introduced at the 2024 Paris Olympics have contributed to improving the overall quality of the Olympics.	0.712	0.046	18.511			
Expectation-disconfirmation	The 2024 Paris Olympics, which introduced new sports, were more novel than expected.	0.547	—	—	0.719	0.569	0.745
	The 2024 Paris Olympics, which introduced new sports, improved the overall quality of the Olympics beyond expectations.	0.582	0.170	9.710			
	The 2024 Paris Olympics, which introduced new sports, were more exciting than expected.	0.558	0.045	12.245			
Satisfaction	Satisfaction with IOC’s decision to introduce new sports to the 2024 Paris Olympic Games.	0.768	—	—	0.821	0.607	0.788
	There is general satisfaction with the new sports introduced at the 2024 Paris Olympics.	0.666	0.049	16.590			
	The 2024 Paris Olympics, featuring newly introduced sports, are considered one of the greatest Olympic events of all time.	0.623	0.111	13.011			
Intention to continue viewing	Consider viewing Olympic sports content regularly.	0.795	—	—	0.869	0.690	0.806
	Given the opportunity, I would view Olympic sports content that introduces new sports.	0.823	0.056	20.286			
	Intend to continue viewing Olympic-related sports content in the future.	0.817	0.056	16.441			

*χ*^2^ = 119.224 (df = 45, *p* = 0.000), CFI = 0.921, NFI = 0.908, TLI = 0.854, RMR = 0.077, and RMSEA = 0.059.

The CFA results were as follows: *χ^2^* = 119.224 (*df* = 45, *p* = 0.000), CFI = 0.921, NFI = 0.908, TLI = 0.854, RMR = 0.077, and RMSEA = 0.059. According to Bagozzi and Dholakia (1994), the best model was evaluated when the CFI, NFI, and TLI were 0.8–0.9 or more, and the RMR and RMSEA were 0.05–0.08 or less. Ultimately, this model satisfied the acceptance level suggested by Bagozzi and Dholakia (1994), indicating that it is a relatively good model. Furthermore, the construct reliability (CR) of all variables was 0.719–0.885, and the average variance extracted (AVE) was 0.569–0.791. This indicated that the suggested fit criteria were as follows: eigenvalue > 0.5, CR > 0.7, and AVE > 0.5. Each variable was found to have convergent validity by satisfying these criteria. Nunnally and Bernstein (1994) explained that there is no problem with reliability if the alpha coefficient is 0.5 or more when the reliability test is conducted for all the questions. As a result of using the internal consistency reliability analysis method with Cronbach’s *α* value for reliability verification (Hair et al., 2010), we obtained Cronbach’s *α* value of 0.745–0.877, indicating relatively high reliability.

### 3.4 Data analysis process

Valid questionnaires were analyzed using the Statistical Package for Social Sciences (SPSS, version 28.0) and Analysis of Moment Structures (AMOS, version 26.0) after the coding and error reviews. First, the general characteristics of the participants were analyzed using frequency analyses. Second, CFA was performed to verify all factors, and reliability was assessed using Cronbach’s *α* coefficient to ensure internal consistency. Third, correlation analysis was performed to analyze the relationships between the variables, and structural equation modeling (SEM) was performed to derive a structural model.

## 4 Results

### 4.1 Correlation analysis

We performed a correlation analysis to confirm the correlations between the variables. No multicollinearity problem was observed because no variable showed a correlation of 0.8 or higher in the range of the correlation coefficient value of 0.386–0.687 (Challagalla and Shervani, 1996) (Table 3). Multicollinearity is a statistical issue that arises when two or more independent variables are highly correlated, potentially leading to unstable estimates of regression coefficients and reduced clarity of interpretation (Hair et al., 2010). We observed no problems in structurally verifying the theoretical model of this study.

**Table 3:** Correlation analysis.

Variables	1	2	3	4	5
Prior expectation^1^	1				
Perceived performance^2^	0.226** (0.051)	1			
Expectation-disconfirmation^3^	0.269** (0.072)	0.376** (0.141)	1		
Satisfaction^4^	0.285** (0.081)	0.344** (0.118)	0.394** (0.155)	1	
Intention to continue viewing^5^	0.458** (0.209)	0.371** (0.137)	0.367** (0.134)	0.351** (0.123)	1

**p < 0.01.

1 Prior expectation.

2 Perceived performance.

3 Expectation-disconfirmation.

4 Satisfaction.

5 Intention to continue viewing, () is the square value of the correlation coefficient.

In addition, a discriminant validity analysis was performed. Discriminant validity refers to the extent to which constructs are empirically distinct from one another and is typically assessed by comparing the square root of the AVE for each construct with the inter-construct correlation coefficients. Fornell and Larcker (1981) suggested that discriminant validity could be secured if the AVE value was larger than the squared value of the correlation coefficient. The largest square value of the correlation coefficient was 0.458 (= 0.209), and the smallest value of the AVE was 0.569, ensuring discriminant validity.

Finally, the potential presence of common method bias (CMB), which can occur in survey-based research, was examined (Higgs et al., 2005). CMB refers to the risk that the relationships among variables may be overestimated or underestimated when data are collected using the same measurement instrument or response format. We employed the single common method factor approach proposed by Podsakoff et al. (2003). This technique involves comparing a CFA model without a common method factor to a model that includes such a factor and examining whether the changes in the factor loadings are substantial. The results indicated that the models with and without the common method factor demonstrated acceptable levels of fit. Moreover, the changes in item loadings were all below 0.20, suggesting that the influence of CMB was not substantial in this study (Ruonan et al., 2023).

### 4.2 Model verification

**Figure 2:**
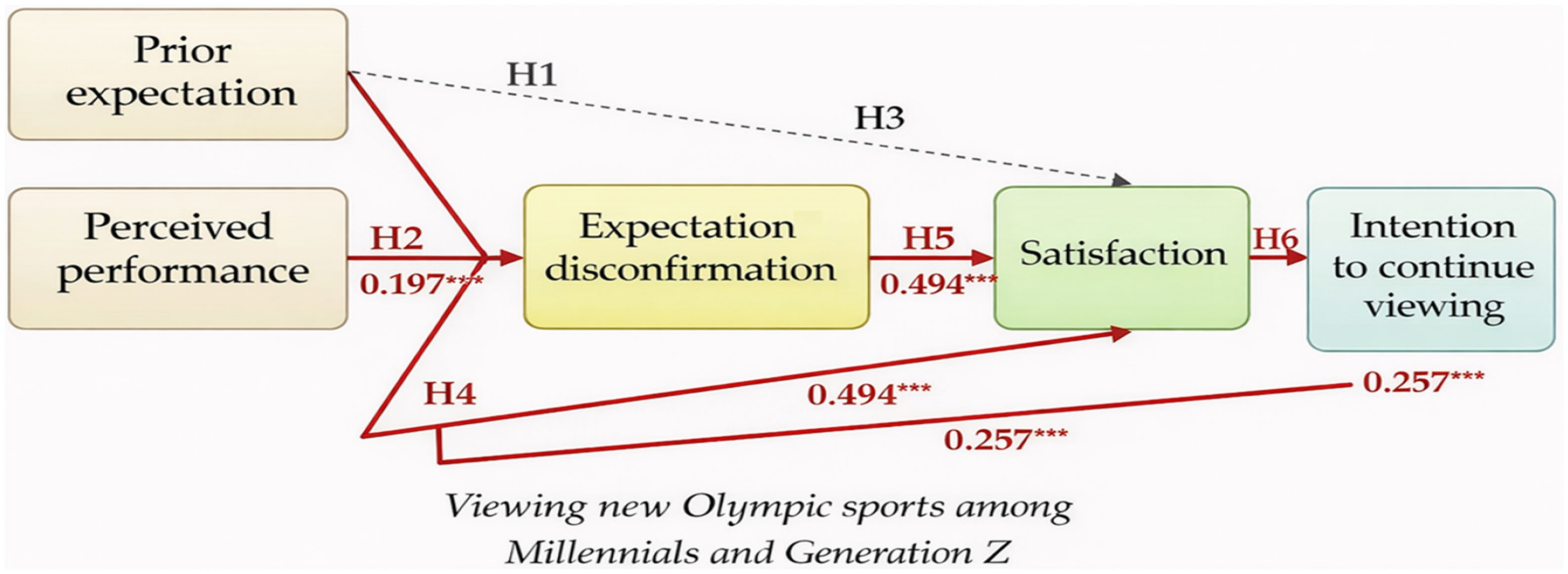
Conceptual framework of the study.

Prior to conducting the SEM analysis, Harman’s single-factor test was performed to rigorously examine the potential influence of the CMB. The results showed that the single factor accounted for 24.9% of the total variance, which is below the recommended threshold of 50% proposed by Podsakoff and Organ (1986). This indicates that CMB is unlikely to pose a serious threat to the validity of the findings in this study. Subsequently, to verify the suitability of the SEM in this study, the structural equation model was analyzed, and the results were as follows: *χ^2^* = 117.047, *df* = 47, CFI = 0.917, NFI = 0.901, TLI = 0.878, RMR = 0.064, and RMSEA = 0.056 (Table 4). The test analysis provided a path coefficient of H1 = 0.114 (*t* = 1.379, *p* > 0.05), indicating that H1 was rejected. Second, the path coefficient of H2 was 0.197 (*t* = 5.691, *p* < 0.001), indicating that H2 was accepted. Third, the path coefficient of H3 was 0.041 (*t* = 5.257, *p* > 0.05), indicating that H3 was rejected. Fourth, the path coefficient of H4 was 0.259 (*t* = 5.257, *p* < 0.001), indicating that H4 was accepted. Fifth, the path coefficient for H5 was 0.494 (*t* = 4.622, *p* < 0.001), indicating that H5 was supported. Finally, the path coefficient for H6 was 0.257 (*t* = 4.172, *p* < 0.001), indicating that H6 was accepted in this study (Figure 2).

**Table 4:** Hypothesis testing results.

H	Path	*SE*	*CR*	*p*	Accept/Reject
H1 Prior expectation → Expectation-disconfirmation	0.114	0.082	1.379	0.168	Reject
H2 Perceived performance → Expectation-disconfirmation	0.197	0.035	5.691	0.000***	Accept
H3 Prior expectation → Satisfaction	0.041	0.030	1.379	0.176	Reject
H4 Perceived performance → Satisfaction	0.259	0.037	5.257	0.000***	Accept
H5 Expectation-disconfirmation → Satisfaction	0.494	0.107	4.622	0.000***	Accept
H6 Satisfaction → Intention to continue viewing	0.257	0.062	4.172	0.000***	Accept

***p < 0.001.

## 5 Discussion

This study empirically analyzed the gap between the expectations and actual experiences of Millennials and Generation Z regarding breaking, skateboarding, surfing, and sport climbing, which were newly introduced as official events in the 2024 Paris Olympics. We applied the EDT, and the following discussion is based on the results.

First, prior expectations did not have a significant effect on expectation disconfirmation, indicating that H1 was rejected. These results may be interpreted as contradicting the theoretical premise of previous studies (International Olympic Committee, 2020a; Tolman, 1945; Oliver, 1997; Oliver, 2010; Tse and Wilton, 1988; Van Dolen et al., 2004), which argued that the prior expectations of Millennials and Generation Z—an emerging key consumer group for future Olympic content—directly influence expectation disconfirmation. This suggests that Millennials and Generation Z are more influenced by actual content experiences and perceived performance than by their fixed prior expectations. In other words, experience-based evaluation factors, such as content quality, interest, and immersion derived from actual viewing, appear to have a more decisive impact on the formation of expectations and satisfaction than prior information or preconceptions about the new Olympic sports. Similarly, this study found that prior expectations did not have a significant effect on satisfaction, indicating that H3 was rejected. These results contradict those of Churchill and Surprenant (1982) and Spreng et al. (1996), who reported that prior expectations had direct and indirect effects on satisfaction. According to Park et al. (2021), for digital native generations such as Millennials and Generation Z, satisfaction is shaped more by direct interaction, immersive experiences, and emotional responses than by passive expectation formation when consuming content. This suggests that cognitive processes, such as perceived performance rather than prior expectations, serve as key determinants of satisfaction. In addition, the new sports analyzed in this study, such as breaking, skateboarding, surfing, and sport climbing, were introduced when public experience with them was relatively limited. Therefore, it is possible that respondents’ expectations were either not clearly formed or highly variable. This may explain the low predictive power of prior expectations and their limited effect on satisfaction (Hien et al., 2024).

Second, perceived performance had a significant effect on expectation disconfirmation, supporting H2. This result aligns with the core assumption of the EDT (Conti, 2023; Kim and Chung, 2025; Kim and Hwang, 2019; Hien et al., 2024), which posits that the level of performance experienced by consumers serves as a key reference point for comparing it with their prior expectations (Marques et al., 2025; Oliver, 1980). In other words, Millennials and Generation Z formed expectation disconfirmation by assessing how much the actual performance of the sports newly introduced as official events in the 2024 Paris Olympics exceeded or fell short of their prior expectations. This disconfirmation directly influenced subsequent behavioral responses such as satisfaction and intention to continue viewing.

Third, prior expectation did not have a significant effect on expectation disconfirmation, indicating that H3 was rejected. The effect of prior expectations on satisfaction was found to be inconsistent with previous studies by Kim et al. (2010) and Parasuraman et al. (1988). This suggests that this study fails to reflect the diversity and heterogeneity of Millennials and Generation Z in relation to new Olympic sports. This finding also confirms that expectation disconfirmation indirectly influenced the satisfaction of Millennials and Generation Z with new Olympic events. Therefore, satisfaction with a new Olympic sport may be substantially shaped by the contrast between limited expectations and rich experiences.

Fourth, perceived performance had a significant effect on satisfaction, supporting H4. This finding corroborates that of Oliver and DeSarbo (1988) and Tse and Wilton (1988) by asserting that perceived performance has a positive effect on satisfaction. In particular, in content types where experiential and immersive engagement is crucial, such as Olympic sports content, perceived performance extends beyond mere functional evaluation to encompass emotional and psychological satisfaction, which plays a decisive role in shaping users’ overall evaluations (Van Dolen et al., 2004). This confirms that perceived performance among Millennials and Generation Z is a reliable predictor of satisfaction with Olympic sports content consumption. This underscores the importance of managing content quality and enhancing services based on user experience in the Olympic sports media industry.

Fifth, expectation disconfirmation had a significant effect on satisfaction, supporting H5. Recent related studies, such as Loureiro et al. (2022), suggest that the more positive the discrepancy between users’ prior expectations and their actual online experiences based on services, the greater the increase in satisfaction. Lee and Jeong (2023) conducted a study on users of digital content platforms. They found that when content quality and personalized experiences exceed expectations, both user satisfaction and reuse intention increase, which is consistent with the results of this study. These results suggest that when Millennials and Generation Z perceive performance in a newly introduced official Olympic sport as exceeding expectations, their emotional responses and cognitive evaluations interact synergistically to enhance overall satisfaction (Li et al., 2020; Pitt et al., 1997).

Finally, satisfaction had a significant effect on the intention to continue viewing, supporting H6. Oliver (1981) stated that satisfaction is not merely a short-term emotional response but also serves as an emotional and cognitive foundation that reinforces the intention for repeated consumption behavior. When consumers experience outcomes that exceed their expectations, they develop high levels of satisfaction through positive disconfirmation, which often translates into content loyalty and continued usage intentions. In addition, Chen et al. (2022) demonstrated that OTT platform users’ satisfaction strongly influences their intention to continue viewing and serves as a key mediating variable in the EDT framework. Similarly, Zhou and Lu (2021) found that satisfaction formed when the immersive experience of sports content exceeded expectations had a significant impact on the intention to rewatch, providing implications consistent with the results of this study (Oliver, 1980; Bang et al., 2019; Chiu et al., 2007; Duarte et al., 2018).

In summary, the results demonstrated that the EDT possesses valid explanatory power in the context of Olympic sports content consumption. When targeting digital native generations such as Millennials and Generation Z, evaluations based on perceived performance and actual experience exerted a stronger and more direct influence on satisfaction and the intention to continue viewing than fixed prior expectations. This suggests that media strategies for mega sporting events, such as the Olympics, should move beyond simple information delivery and prioritize user-centered immersive experience design, enhanced content quality, personalized interfaces, and increased interactivity as key strategic elements. Therefore, when public awareness of newly introduced Olympic sports is low and expectations are unclear, sophisticated planning is required for content formatting, production, and integration with digital platforms. This is because the quality of the viewing experience and the accompanying emotional response play critical roles in shaping user satisfaction and driving repeated engagement.

### 5.1 Limitations

This study had some limitations. First, this study focused solely on Millennials and Generation Z residing across 17 cities and provinces in South Korea, limiting the regions and age range of the sample. Consequently, cultural differences across countries, variations in access to Olympic sports content, and differences in media usage environments across regions were not considered in this study. These factors may limit the generalizability of our findings. Therefore, future research should include a more diverse and representative sample to enable broader and more comprehensive analyses. Second, this study was based on quantitative research utilizing survey data, which presents limitations in capturing temporal changes in expectations and satisfaction and cognitive changes that may occur through repeated viewing experiences. Therefore, future research should adopt a mixed-methods approach that incorporates qualitative research methods, such as interviews, to more precisely examine the causal relationships between expectations, perceived performance, satisfaction, and behavioral intentions.

## 6 Conclusion

This study applied the EDT to examine the gap between prior expectations and actual viewing experiences of newly introduced Olympic sports (breaking, skateboarding, surfing, and sport climbing) among Millennials and Generation Z in the context of the 2024 Paris Olympics. The results generally supported the core logic of the expectancy theory and showed generational and situational differences in satisfaction and the formation of behavioral intentions.

Prior expectations did not significantly affect expectation disconfirmation or satisfaction. This suggests that for novel sports with limited public familiarity, expectations may be weakly formed or highly heterogeneous, thereby reducing their explanatory power. In contrast, perceived performance emerged as a key determinant of expectation disconfirmation and satisfaction, highlighting the importance of actual experiential quality over preexisting beliefs. Furthermore, expectation disconfirmation had a significant positive effect on satisfaction, indicating that experiences that exceed vague or uncertain expectations can elicit strong emotional and cognitive responses. Finally, satisfaction significantly influenced the intention to continue viewing, confirming its central role in shaping the long-term engagement with new Olympic sports.

From a theoretical perspective, this study extends the EDT to the context of the newly introduced Olympic sports. This demonstrates that, for digital native generations, satisfaction is driven more by experience-based evaluations than by stable prior expectations. This finding challenges the traditional emphasis on the centrality of expectations in the EDT and underscores the importance of perceived performance and experiential immersion in the formation of satisfaction. Moreover, this study enables more sophisticated theoretical extensions of existing EDT mechanisms by empirically demonstrating that the relative influence of EDT components can be rebalanced in novel content environments with high levels of uncertainty.

From a practical standpoint, the findings suggest that strategies for promoting new Olympic sports should prioritize the quality of actual viewing experiences instead of relying solely on pre-event promotions or expectation-building campaigns. For Millennials and Generation Z, immersive storytelling, emotional engagement, interactivity, and platform usability are crucial for shaping satisfaction and sustaining long-term viewing intentions. In particular, when public awareness of new sports is low, experience-centered content design becomes a decisive factor in fostering continued engagement.

In conclusion, this study reaffirms the explanatory power of the digital-native generation in sports media consumption while highlighting the need for experience-centered strategies to attract and retain digital-native viewers. These insights have meaningful implications for the long-term diffusion and sustainability of newly introduced Olympic sports.

## Data Availability

The original contributions presented in the study are included in the article/supplementary material, further inquiries can be directed to the corresponding authors.
